# Abscisic acid transporters cooperate to control seed germination

**DOI:** 10.1038/ncomms9113

**Published:** 2015-09-03

**Authors:** Joohyun Kang, Sojeong Yim, Hyunju Choi, Areum Kim, Keun Pyo Lee, Luis Lopez-Molina, Enrico Martinoia, Youngsook Lee

**Affiliations:** 1 Institut für Pflanzenbiologie, Universität Zürich, Zollikerstrasse 107, 8008 Zürich, Switzerland; 2 POSTECH-UZH Global Research Laboratory, Division of Molecular Life Sciences, Pohang University of Science and Technology, Pohang 790-784, Korea; 3 Départment de Biologie Végétale, Université de Genève, 1211 Geneva 4, Switzerland; 4 Division of Integrative Biology and Biotechnology, POSTECH, Pohang 790-784, Korea

## Abstract

Seed germination is a key developmental process that has to be tightly controlled to avoid germination under unfavourable conditions. Abscisic acid (ABA) is an essential repressor of seed germination. In *Arabidopsis*, it has been shown that the endosperm, a single cell layer surrounding the embryo, synthesizes and continuously releases ABA towards the embryo. The mechanism of ABA transport from the endosperm to the embryo was hitherto unknown. Here we show that four AtABCG transporters act in concert to deliver ABA from the endosperm to the embryo: AtABCG25 and AtABCG31 export ABA from the endosperm, whereas AtABCG30 and AtABCG40 import ABA into the embryo. Thus, this work establishes that radicle extension and subsequent embryonic growth are suppressed by the coordinated activity of multiple ABA transporters expressed in different tissues.

## Abstract (web summary)

Seed germination is repressed by release of the plant hormone abscisic acid (ABA) to the embryo from the surrounding endosperm tissue. Here Kang *et al*. characterize four different ABA transporters and propose that they act in concert to control ABA release and regulate germination.

## Graphical abstract

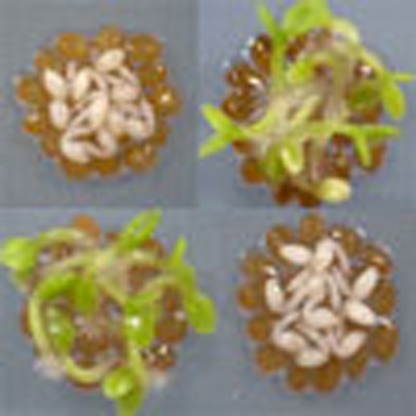

Seed germination is tightly controlled in plants. *Arabidopsis* seeds exhibit primary dormancy, a trait whereby germination of freshly produced seeds is blocked even under favourable germination conditions^1^,^2^. Many studies have sought to decipher the complex regulatory network that controls seed germination, and it is apparent that abscisic acid (ABA) and gibberellins (GAs) antagonistically regulate seed germination in both dormant and non-dormant seeds^3^,^4^,^5^,^6^,^7^. Whereas ABA represses seed germination, GAs promote it^2^,^8^,^9^. In non-dormant seeds, environmental parameters, such as light quality, temperature or water potential, determine the endogenous production of ABA and GA^10^,^11^,^12^. Recent work has shown that GAs promote germination by negatively regulating ABA levels^13^, which stimulate the production of germination repressors, such as transcription factors ABI3 and ABI5 (refs 12, 13, 14, 15). GA promotes the proteasome-mediated destruction of DELLA factors that promote endogenous ABA accumulation, leading to seed germination^12^,^13^.

Dormant seeds typically block germination by maintaining high levels of ABA on imbibition^16^. Accordingly, mutant seeds unable to synthesize ABA lack dormancy; that is, they germinate precociously and may even germinate while still attached to the mother plant^17^. This phenomenon, termed vivipary, results in economic loss in crop species. *Arabidopsis* natural accessions display markedly different dormancy levels: the *Cape Verde Island* (Cvi) ecotype exhibits much stronger dormancy at harvest than the *Columbia-0* (Col-0) or *Landsberg erecta* (L*er*) ecotypes^18^,^19^. These ecotypes differ in ABA content in the dry seed and the speed of ABA degradation on imbibition^20^. Quantitative trait loci involved in the delay of germination have been identified by comparing the Cvi and L*er* ecotypes^21^,^22^, and some of the loci have been suggested to be related to ABA signalling or GA sensitivity^21^,^22^,^23^.

One intriguing and poorly understood aspect of seed physiology is the spatial and temporal regulation of ABA transport on seed imbibition. The *Arabidopsis* seed consists of an embryo, an active endosperm layer surrounding the embryo, and an outer layer of dead tissue, the testa, of maternal origin^24^. A former study indicated that in dormant seeds, ABA is produced by both the embryo and endosperm^25^,^26^,^27^,^28^. More recent studies revealed that some inhibitory compounds produced by tissues surrounding the embryo are responsible for dormancy^29^,^30^,^31^. Elegant experiments conducted by Bethke *et al*.^25^ further showed that the endosperm layer is responsible for most, if not all, of the germination-repressive activity in *Arabidopsis*. Recently, Lee *et al*.^32^ developed a seed coat bedding assay (SCBA) that allows the monitoring of growth of dissected embryos cultured on a layer of seed coat bedding, which consists of testa and endosperm. This assay is useful to identify components active in either endosperm or embryo that control germination. Indeed, this assay provided direct evidence that ABA is released from the endosperm of dormant seeds on imbibition to inhibit embryo development. The SCBA was used to further show the importance of the endosperm in non-dormant seeds. An early far-red light pulse that blocks germination involves phytochrome B (phyB) inactivation in the endosperm, which blocks the expression in GA biosynthesis genes in the endosperm, and induces ABA release towards the embryo^33^. Furthermore, recent work suggested that in *ga1* seeds, which are unable to synthesize GA, the endosperm also releases ABA to block germination^34^.

However, how ABA is transported between the endosperm and embryo in seeds remains poorly understood. Until recently, it was assumed that ABA, being a weak acid (p*K*_a_=4.9), passes through biological membranes via passive diffusion when protonated, and is then trapped in the dissociated form in the neutral pH environment of the cytosol^35^. However, auxin, another weak acid and a potent growth-promoting phytohormone, is not only transported by passive diffusion but also by different types of transporters, which enables the plant to respond accurately and rapidly to environmental changes by adjusting its growth^36^,^37^,^38^. Thus, it is tempting to speculate that a multitude of ABA transporters coordinately function to deliver ABA to specific tissues in response to environmental and developmental cues. Indeed, recent studies identifying several ABA transporters might be a prelude to the discovery of such a network of ABA transporters^39^,^40^,^41^,^42^,^43^,^44^. Two ABC transporters of the ABCG subfamily, AtABCG25 and AtABCG40, export ABA from the vascular bundle and import it into guard cells, respectively^39^,^40^. Furthermore, another ABC transporter, AtABCG22, was proposed to function as an ABA transporter^41^. In addition, several members of the ABA-importing transporter family function as ABA importers at the influorescence stem^42^,^43^. Recently, a MATE-type transporter has also been reported to act as an ABA efflux carrier in guard cells^44^.

We reasoned that ABA delivery from the endosperm to the embryo must be accurately controlled and thus might be mediated by multiple coordinated groups of transporters, rather than by diffusion alone. We searched for candidate transporters among the ABCG subfamily of ABC proteins, since this family mediates the efflux and influx of a wide variety of terpenoids^45^, including ABA.

Here we report that four AtABCG proteins function together to deliver ABA from the endosperm to the embryo in mature, imbibed seeds. AtABCG25 and AtABCG31 are mainly localized to the endosperm and export ABA from the endosperm to the embryo, whereas AtABCG30 and AtABCG40 are localized to the embryo and transport ABA into the embryo. ABA transport represents an important step in embryo germination and early seedling establishment. Thus, this work demonstrates that a developmental step critical for a plant's survival is efficiently regulated by the coordinated activity of multiple ABA transporters localized in different tissues.

## Results

### Screening for candidate ABA transporters in seeds

**Figure 1:**
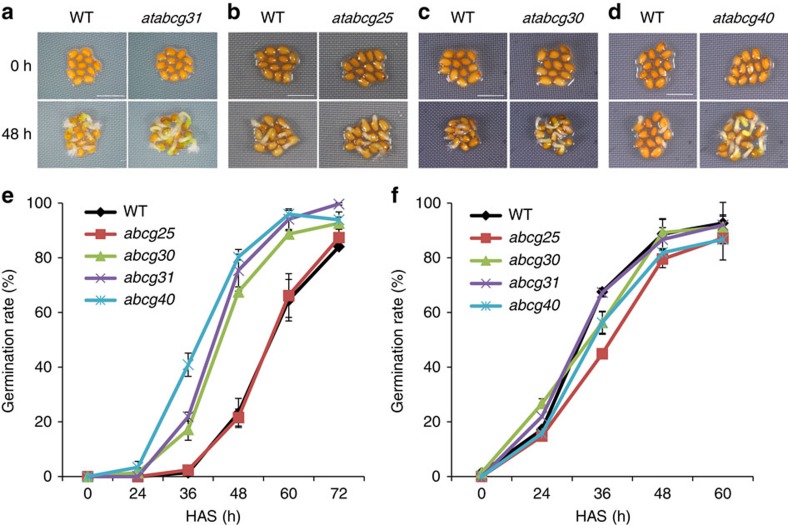
Seeds of *atabcg31*, *atabcg30* and *atabcg40* germinate faster than those of the corresponding wild type (WT). (**a**–**d**) Seed germination on ½ MS medium without stratification*. atabcg31* (**a**), *atabcg30* (**c**) and *atabcg40* (**d**) mutant seeds germinated faster than the WT, whereas *atabcg25* mutant seeds (**b**) did not. WT seeds collected at the same time were used as the control. Photographs were taken at the start of incubation (0 h) and 48 h later. A representative result out of three independent experiments giving rise to similar results is shown. Each experiment consisted of two or three replicates. Scale bar, 1.25 mm (**a**), 1 mm (**b**–**d**). (**e**,**f**) Germination rate of WT seeds compared with that of *atabcg* mutant seeds on ½ MS medium. Non-stratified (**e**) or stratified (**f**) seeds were sowed on ½ MS medium, and germinated seeds were determined based on radicle extrusion. Stratification consisted of pretreatment of seeds for 2 days at 4 °C in the dark. In all experiments, fresh seeds were used, and the sowed seeds were incubated under 16-h light and 8-h dark conditions at 22 °C. Data are mean±s.e.m. of three independent experiments with four technical replicates (*N*=3, *n*=4 × 100 seeds). HAS, hours after sowing.

To select candidate ABA transporters that regulate seed germination, we first performed an *in silico* analysis (*Arabidopsis* eFP browser; http://bar.utoronto.ca/efp/cgi-bin/efpWeb.cgi) of all 43 members of the ABCG subfamily of ABC transporters, and identified 10 genes that are highly expressed in mature seeds. To determine whether any of these candidates may function as ABA transporters within seeds, we screened the mutants for altered patterns of seed germination (Fig. 1). Six of these ten lines did not have an altered germination phenotype. In contrast, the mutant lines of two candidate genes, *AtABCG30* and *AtABCG31*, exhibited clear differences in germination when compared with wild-type seeds. Seeds from freshly collected *atabcg31* and *atabcg30* mutants germinated earlier than the corresponding wild-type seeds on imbibition without stratification (Fig. 1a,c,e), whereas seed stratification abolished the difference between the mutants and wild type (Fig. 1f). To further investigate whether *atabcg31* and *atabcg30* mutant seeds exhibit deficiencies in ABA transport, we compared their responses with exogenously added ABA and found that germination of stratified *atabcg31* and *atabcg30* seeds was less inhibited by 0.1–1.0 μM ABA than was that of the corresponding wild-type seeds (Supplementary Fig. 1a–e). Similarly, we examined whether *AtABCG25* and *AtABCG40* function as ABA transporters in seeds. Indeed, *atabcg40* seeds exhibited a higher germination rate than the wild type on medium containing 0.1 μM ABA (Supplementary Fig. 1b), suggesting that ABA uptake was reduced in these seeds, consistent with a previous report^40^. Furthermore, seeds from freshly collected, non-stratified *atabcg40* mutants also germinated earlier than those from the corresponding wild type (Fig. 1d,e). In contrast, the germination of non-stratified, freshly collected *atabcg25* seeds was similar to that of wild-type seeds (Fig. 1b,e), even when grown on medium containing exogenous ABA (Supplementary Fig. 1f). This finding contradicts a recent report that showed that the germination of *atabcg25* seeds is delayed on medium containing ABA and that AtABCG25 acts as an ABA exporter^39^. This discrepancy may be explained by the ecotype difference and mild nature of the mutant's phenotype, which may depend on plant growth conditions. Given the convincing data presented previously^39^, we continued to include *atabcg25* in our studies. Taken together, these results strongly suggest that at least four AtABCG transporters are involved in controlling seed germination.

### *AtABCGs* are expressed either in the embryo or endosperm

**Figure 2:**
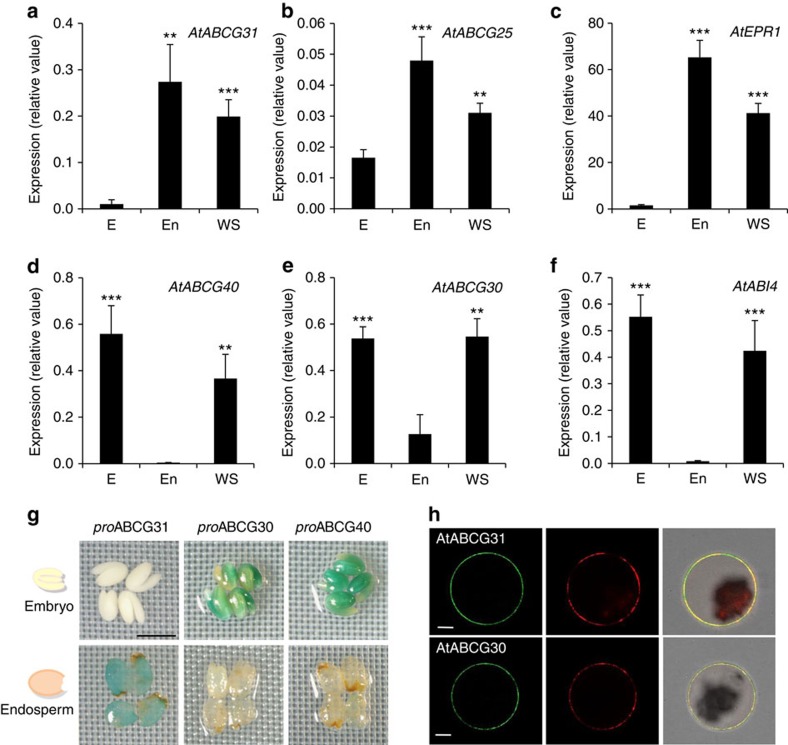
*AtABCG31* and *AtABCG25* are expressed mainly in the endosperm, whereas *AtABCG30* and *AtABCG40* are expressed specifically in the embryo. (**a**–**f**) q-PCR analysis of *AtABCG31* (**a**); *AtABCG25* (**b**); *AtEPR1*, a gene specifically expressed in the endosperm (**c**); *AtABCG40* (**d**); *AtABCG30* (**e**); and *AtABI4*, a gene specifically expressed in the embryo (**f**). q-PCR was performed using total RNA extracted from dissected embryos or endosperms, or whole seeds of the Col-0 wild type. Data were normalized using *AtUbiquitin11*. E, dissected embryos; En, dissected endosperms; WS, whole seeds. Data are mean±s.e.m. of three independent experiments (*N*=3, *n*=2). (**P<0.01, ***P<0.005; (**a**,**b**,**c**) compared with the embryo (E) and (**d**,**e**,**f**) compared with the endosperm (En) by Student's *t*-test). (**g**) *AtABCG31* promoter–GUS (*pAtABCG31::uidA*) reporter gene activity was detected only in the endosperm, and not in the embryo (left). *AtABCG30* promoter–GUS (*pAtABCG30::uidA*) and *AtABCG40* promoter–GUS (*pAtABCG40::uidA*) activities were detectable only in the embryo and not in the endosperm (middle and right). Embryos (upper panel) and endosperms (lower panel) were dissected from mature seeds and incubated in GUS solution for 30 min. Results representative of 8 (*AtABCG30*), 12 (*AtABCG31*) and 9 (*AtABCG40*) different T3 promoter–GUS lines. Scale bar, 0.8 mm. (**h**) Plasma membrane localization of AtABCG31 and AtABCG30 in *Arabidopsis* mesophyll protoplasts. Protoplasts were isolated from *Arabidopsis* leaves transformed with *p35S*::sGFP::*AtABCG31* (upper panels) or *p35S*::*AtABCG30*::GFP (lower panels) and *pd35S*::*AtPIP2A*::mCherry (PM-rK) using polyethylene glycol transformation. The left panel shows the GFP fluorescence of ABCGs, the middle panel the red mCherry fluorescence of the plasma membrane marker PM-rK protein and the right panel shows the merged images of GFP, mCherry and bright-field. Scale bar, 10 μm. GUS, β-glucuronidase.

To further explore whether these AtABCG proteins indeed influence germination by regulating ABA transport within the seed on imbibition, we analysed the transcript levels of the corresponding genes in the endosperms and embryos of mature wild-type seeds. Tissue-specific quantitative reverse transcription–PCR (qRT–PCR) revealed that *AtABCG31* was not expressed in dissected embryos, but was expressed at high levels in the endosperm (Fig. 2a). *AtABCG25* was mainly expressed in the endosperm and, to a lesser extent, in the embryo (Fig. 2b). This result is consistent with previous *in silico* data^31^. In contrast, *AtABCG30* and *AtABCG40* transcripts were strongly expressed in dissected embryos, but only weakly in the endosperm (Fig. 2d,e). To validate our approach, we performed qRT–PCR with two genes known to be expressed either in the endosperm (*AtEPR1*, an extensin-like gene involved in seed germination)^46^ or the dissected embryo (*AtABI4*, encoding an ABA-response transcription factor)^31^. Consistent with previous reports, *AtEPR1* transcripts were detected solely in the endosperm, whereas *AtABI4* transcripts were detected nearly exclusively in the embryo (Fig. 2c,f). Our analysis of multiple lines of promoter–β-glucuronidase-expressing plants (Fig. 2g and Supplementary Fig. 2a) confirmed the qRT–PCR results; the *AtABCG31* promoter was active only in the endosperm layer, and β-glucuronidase activity driven by the *AtABCG30* and *AtABCG40* promoters was observed in dissected embryos, but not in the endosperm. Together, these results show that *AtABCG31* and *AtABCG25* expression is specific to the endosperm, where their protein products may act as ABA efflux transporters. In contrast, *AtABCG30* and *AtABCG40* expression is specific to the embryo, where their protein products may function as ABA influx transporters. To function as transporters that facilitate ABA flux from the endosperm to the embryo, these four transporters are expected to be localized to the plasma membrane. AtABCG25 and AtABCG40 were previously shown to localize to the plasma membrane^39^,^40^,^47^. Here we show that AtABCG31 and AtABCG30 are also localized to the plasma membrane of *Arabidopsis* mesophyll protoplasts and tobacco epidermis cells when transiently expressed under the control of the 35S promoter (Fig. 2h and Supplementary Fig. 2b). Co-expression of green fluorescent protein (GFP)-fused AtABCG31 or AtABCG30 with PM-rK (ref. 48), a plasma membrane marker protein (an mCherry fusion of the plasma membrane aquaporin, AtPIP2A), showed complete co-localization of the two ABCG proteins with PM-rK. Plasma membrane localization of AtABCG31 was recently also shown by Choi *et al*.^49^.

### *ABCG31* and *ABCG25* repress embryonic growth

**Figure 3:**
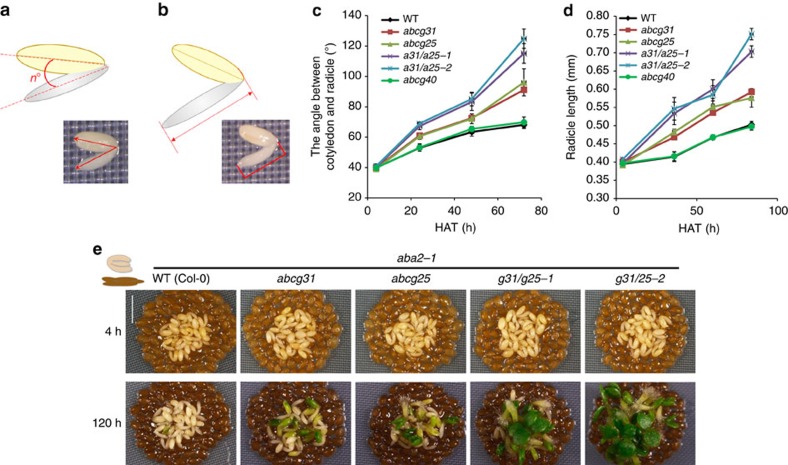
Seed coats of *atabcg31* and *atabcg25* repressed the embryonic growth of *aba2-1* less effectively than did those of the wild type (WT). (**a**,**b**) Diagram showing the quantification of the angle between the radicle and the cotyledon and the radicle length, respectively. (**a**) The angle between the radicle and cotyledon was measured using Axio. (**b**) Radicle length was measured using Axio. (**c**–**e**) Embryos dissected from *aba2-1* mutant seeds were placed on a layer of seed coat beddings dissected from the WT (Col-0), *atabcg31*, *atabcg25*, *atabcg31 atabcg25* (*g31/g25-1, g31/g25-2*), and *atabcg40*. The legend in the graph indicates the genotypes of the seeds from which the seed coat beddings were derived. The angle between the cotyledon and radicle (**c**) and the radicle length (**d**) of the *aba2-1* embryos were measured as indicators of embryonic growth. (**c**,**d**) Data are means±s.e.m. of *n*=108–112 obtained from three independent experiments. Each experiment was performed in duplicate. Each replicate (one SCB) consisted of 20 embryos (*N*=3, *n*=2 × 20 embryos). (**e**) Photographs taken 4 h (top) and 120 h (bottom) after imbibition. Scale bar, 1.25 mm. HAT, hours after transfer. Results shown are representative of three independent experiments (see Supplementary Fig. 7). Each experiment was performed in duplicate. Similar results were obtained in all experiments.

We next sought to determine whether the embryo or endosperm is responsible for the altered germination phenotype of mutant seeds by performing SCBAs as described by Lee *et al*.^32^,^33^. SALK insertion *Arabidopsis* lines are generated in the Col-0 ecotype, which displays relatively weak and short-term dormancy compared with ecotypes such as L*er* or Cvi, for which the SCBA was initially used. As a result, a SCBA using freshly collected Col-0 material may result in less robust embryonic growth arrest results. To better test the role of *AtABCG31* and *AtABCG25* in controlling embryonic growth, we performed SCBAs in presence of paclobutrazol (PAC), a GA synthesis inhibitor. Lack of GA synthesis promotes ABA accumulation and efficiently blocks embryonic growth in SCBAs^13^,^34^,^50^. Indeed, in presence of PAC, seed coats of Col-0 prevented embryonic growth (Supplementary Fig. 3a), and expressed the germination repressor *AtABI5* (Supplementary Fig. 3b)^15^, as effectively as those of Cvi. However, consistent with previous results, PAC did not affect the embryonic growth of dissected embryos or their ability to establish seedlings^9^ (Supplementary Fig. 3c). In this assay, we also used dissected embryos of the *aba2-1* mutant, which is defective in ABA biosynthesis and consequently germinates precociously^51^. For quantitative analysis, we measured the angle between cotyledons and radicles as well as radicle length (Fig. 3a,b), as indicators of early embryonic growth. As shown in Fig. 3, seed coats (testa and endosperm) of the *atabcg31* or *atabcg25* single knockouts did not inhibit the growth or early seedling establishment of the *aba2-1* embryo as effectively as did those of the wild type in the SCBA. Our findings for the *atabcg25* seed coat are inconsistent with our observation that the germination rate of *atabcg25* whole seeds is similar to that of the wild type (Fig. 1b,e and Supplementary Fig. 4a). This inconsistency may be due to a defect in *atabcg25* embryos, which exhibit retarded embryonic growth even when dissected out from the surrounding tissues (Supplementary Fig. 4b). The retarded growth of embryos seems to cancel out the weak inhibitory effect of the endosperm on germination; thus, no aberrations in whole-seed germination are apparent. Reciprocal crossing of *atabcg25* with *atabcg31* yielded *g31/g25-1* (*atabcg31* pistil; *atabcg25* pollen) and *g31/g25-2* (*atabcg31* pollen; *atabcg25* pistil) double mutants, which lack both ABC transporters. The seed coats and endosperms of the double knockouts (*g31/g25-1* or *g31/g25-2*) inhibited the growth and seedling establishment of *aba2-1* embryos much less effectively than did those of the single knockouts or the wild type (Fig. 3c–e and Supplementary Fig. 7). These results suggest that *atabcg31* and *atabcg25* endosperms release reduced amounts of germination inhibitor. In contrast, the seed coat bedding of the other candidate ABC transporter mutants, *atabcg30* and *atabcg40*, did not differ from those of the wild type in terms of their effect on the development of *aba2-1* mutant embryos (Supplementary Fig. 5a, and Fig. 3c,d), indicating that the amount of inhibitor molecules released from the seed coat beddings of wild-type, *atabcg30* and *atabcg40* plants was similar.

### *atabcg30* and *atabcg40* embryos are impaired in ABA uptake

**Figure 4:**
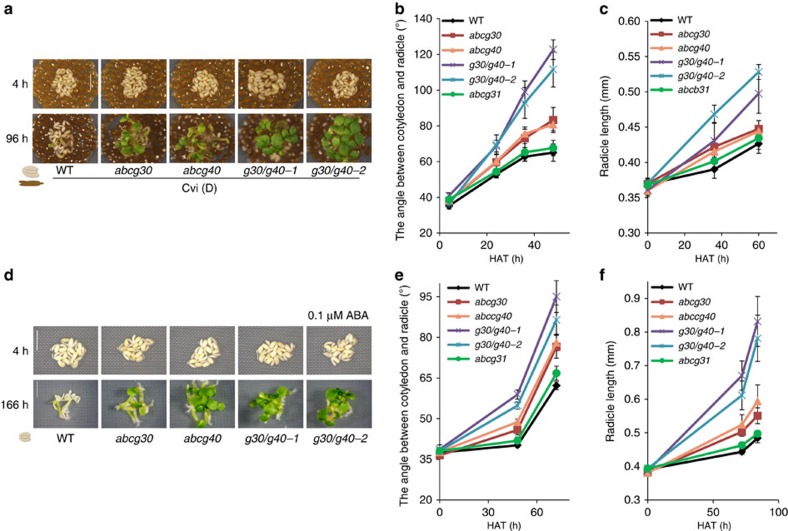
Dissected embryos of *atabcg30*, *atabcg40* and *atabcg30 atabcg40* are less sensitive than the wild type (WT) to endosperm-dependent or exogenous ABA-dependent germination repression. (**a**–**c**) Seed coat bedding assay, using embryos dissected from WT (Col-0) or *atabcg* mutant seeds placed on a layer of seed coat beddings (endosperm and testa) dissected from dormant Cvi seeds (Cvi (D)). (**b**,**c**) Data are means±s.e.m. of *n*=105–109 obtained from three independent experiments. Each experiment was performed in duplicate. Each replicate (one SCB) consisted of 20 embryos (*N*=3, *n*=2 × 20 embryos). (**d**–**f**) Embryos dissected from WT (Col-0) and *atabcg* mutant seeds placed on ½ MS medium supplemented with 0.1 μM ABA. The angle between the cotyledon and radicle (**b**,**e**) and the radicle length (**c**,**f**) were measured as indicators of embryonic growth for each genotype of embryo. (**e**,**f**) Data are means±s.e.m. of *n*=108 obtained from three independent experiments. Each experiment was performed in triplicate. Each replicate consisted of 12 embryos (*N*=3, *n*=3 × 12 embryos). In **a**, photographs were taken at 4 h (top) and 96 h (bottom) after imbibition. In **d**, photographs were taken 4 h (top) and 166 h (bottom) after imbibition. Scale bar, 1 mm. (**a**,**d**) A representative result out of three independent experiments is shown (six (**a**) or nine (**d**) different samples). In all experiments, similar results were obtained.

We next sought to determine whether *AtABCG30* and *AtABCG40* are necessary for embryonic uptake of endospermic ABA. For this purpose, we tested whether *atabcg30* and *atabcg40* mutants are defective in the embryonic uptake of ABA. We evaluated the growth of *atabcg30* (*g30*), *atabcg40* (*g40*) and *g30*/*g40* embryos in SCBAs using seed coats isolated from the highly dormant Cvi ecotype, which were previously shown to efficiently block wild-type embryonic growth in SCBAs^32^. While most embryos of Col-0 wild-type seeds did not grow until 85 h after imbibition (Supplementary Fig. 5a), the embryos dissected from the seeds of *atabcg30* and *atabcg40* and the corresponding double-knockout plants (*g30/g40*) grew and developed to plantlets (Supplementary Fig. 5a and Fig. 4a–c). Thus, these observations indicate that the mutations in *AtABCG30* and *AtABCG40* genes could not efficiently uptake endospermic ABA released by Cvi seed coats relative to wild-type embryos. We further investigated this possibility by monitoring the development of dissected *atabcg30* and *atabcg40* embryos in the absence or presence of exogenous ABA. Under control conditions, no difference in the development of embryos was observed between the wild-type and mutant embryos (Supplementary Fig. 5b); however, in the presence of ABA, the development of embryos dissected from *atabcg30*, *atabcg40* and *g30/g40* double-mutant seeds was inhibited to a lesser extent than those dissected from the wild type (Fig. 4d–f). These observations further indicate that *atabcg30* and *atabcg40* embryos do not take up the ABA released from the seed coat bedding of the Cvi ecotype as effectively as does the wild type.

### Four AtABCGs mediate ABA transport in seeds

**Figure 5:**
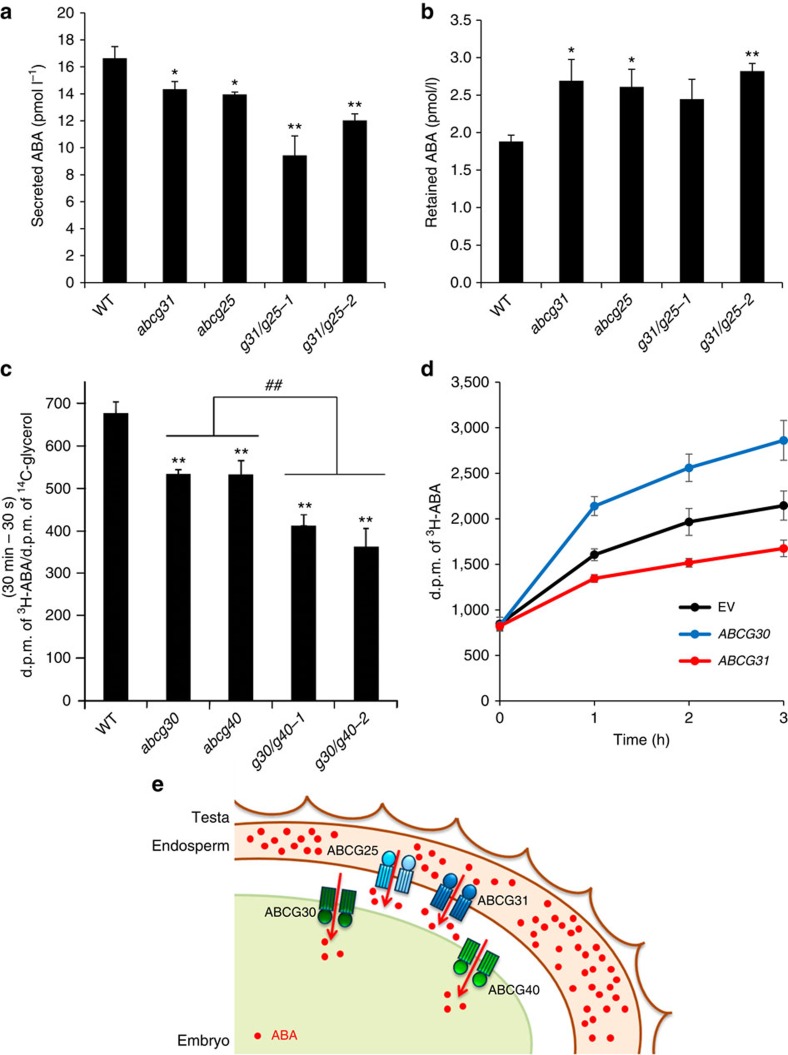
Four AtABCGs mediate ABA transport in *Arabidopsis* seeds. (**a**,**b**) The endosperms and testa of *atabcg* mutants release less ABA than do those of the wild type (WT). Endosperms attached to testa were dissected from 500 WT, *atabcg31*, *atabcg25* and *atabcg31 atabcg25* (*g31/g25-1*, *g31/g25-2*) seeds 24 h after imbibition on medium containing 10 μM PAC. The tissues were then incubated on ½ MS liquid medium for 24 h before ABA content was assayed using an ELISA. The aqueous medium was collected for assay of secreted ABA (**a**), and the remaining tissues were washed three times and extracted to assay the retained ABA (**b**). (**c**) *atabcg30*, *atabcg40* and two different lines of double mutant embryos (*g30/g40-1* and *g30/g40-2*) accumulated less ABA than the corresponding WT. Accumulation of ^3^H-ABA by embryos dissected from WT and mutant seeds during a 30-min period. Embryos were incubated in a solution of 12.5 nM ^3^H-ABA (1.63 Tbq mmol^−1^) and 17.5 pM ^14^C-glycerol (5.40 GBq mmol^−1^) at pH 6.5. The radioactivity uptake was normalized to the ^14^C-glycerol d.p.m. (disintegrations per min) value. (**d**) Time-dependent loading assay of ^3^H-ABA to yeast cells expressing AtABCG31 or AtABCG30, or transformed with the empty vector (EV). Yeast cells were incubated in SG-URA medium containing 50 nM ^3^H-ABA (7.4 kBq, 1.63 Tba mmol^−1^) at pH 6.5. Data are means±s.e.m. of *n*=12 from three independent experiments (*N*=3, *n*=4). (**e**) Four AtABCGs participate in the delivery of ABA from the endosperm to the embryo in *Arabidopsis* seeds. AtABCG31 and AtABCG25 are expressed in the endosperm and secrete ABA from the endosperm to the embryo. AtABCG30 and AtABCG40 are ABA uptake transporters located in the embryo. To inhibit embryo germination, *de novo* biosynthesized ABA is transported from the endosperm to the embryo by the four transporters. Solid arrows indicate the direction of AtABCG-mediated ABA transportation. Data are means±s.e.m. of *n*=6 (**a**,**b**) or *n*=4 (**c**) from three or four independent experiments. (*P<0.05; **P<0.01 compared with the WT by Student's *t*-test and ^##^*P<0.01* compared with the single mutants by Student's *t*-test).

To directly test whether AtABCG31 and AtABCG25 release ABA from the tissues surrounding the embryo, we removed embryos from the seeds and compared the remaining tissues for secreted and retained endogenous ABA levels. As shown in Fig. 5a,b (with PAC) and Supplementary Fig. 6c,d (without PAC), the tissues surrounding the embryos of *atabcg31* and *atabcg25* single and double mutants secreted less and retained more ABA. On the basis of these results and previous studies that found that endospermic ABA inhibits germination^25^,^29^,^30^,^31^,^32^,^33^, we conclude that AtABCG31 and AtABCG25 contribute to the secretion of ABA from the endosperm. It is interesting that the total ABA content did not differ between the wild type and *atabcg31* or *atabcg25* (Supplementary Fig. 6e), further indicating the importance of endospermic ABA release to control embryonic growth.

Next, we incubated embryos dissected from the wild type, the *atabcg30* and *atabcg40* single mutants, and the corresponding double mutants with radiolabelled ABA and determined the amount of radioactivity taken up after 30 min at room temperature. Both mutants took up significantly less ABA than the wild type, and the double-knockout mutants took up less than the single knockouts (Fig. 5c). In contrast, embryos incubated in the presence of radiolabelled ABA on ice did not differ in their ABA contents (Supplementary Fig. 6a). Therefore, the difference in ABA uptake shown in Fig. 5c was mediated by an active transport mechanism and not by simple diffusion. Taken together, these observations further strengthen and lend direct support to the notion that AtABCG30 and AtABCG40 function as endospermic ABA importers. While ABA transport activity has been reported for AtABCG25 and AtABCG40 in heterologous systems^39^,^40^, it has not been reported for AtABCG30 and AtABCG31. Therefore, to obtain additional evidence that these transporters also catalyse ABA fluxes, we expressed these AtABCGs in yeast and performed ABA transport experiments using the yeast system. Yeast expressing AtABCG31 took up lower amounts of ABA than yeast transformed with an empty vector, while yeast expressing AtABCG30, which was localized to the plasma membrane (Supplementary Fig. 6f), accumulated far more radiolabelled ABA (Fig. 5d). These results are in line with those obtained with embryos and seed coats from the corresponding mutants, indicating that AtABCG31 acts as an exporter, while AtABCG30 catalyses the import of ABA.

## Discussion

In this study, we characterize two novel ABA transporter candidates, AtABCG31 and AtABCG30 (Fig. 5d), and provide various lines of evidence that the ABC transporters AtABCG31 and AtABCG25 mediate ABA secretion from the endosperm, and that AtABCG30 and AtABCG40 mediate ABA uptake into the embryo. Interestingly, although all these AtABCG transporters exhibit a common substrate transport activity, they are structurally different and not closely related in phylogenetic tree. AtABCG25 is a half-size ABC transporter, whereas AtABCG31, AtAtABCG30 and AtABCG40 are full-size ABC proteins^52^. Moreover, the three full-size transporters belong to different phylogenetic clusters^52^.

We found that *AtABCG31* transcripts were present in the endosperm (Fig. 2a), which is consistent with a recent report^53^. Freshly collected intact *atabcg31* seeds exhibited an enhanced germination phenotype in the presence (Supplementary Fig. 1c,d) or absence (Fig. 1a) of exogenous ABA. Thus, AtABCG31 appears to function as an efflux transporter that transports ABA from the endosperm to the embryo to prevent germination under unfavourable environmental conditions. AtABCG31 was previously reported to be expressed specifically in guard cells^54^ and the tapetum^49^. ABA plays an important role in both cell types, functioning in stomatal closure and pollen maturation, respectively; however, no direct link to ABA transport was demonstrated.

AtABCG25 was previously reported to function as an ABA exporter that was active mainly in ABA-synthesizing tissues^39^. Here we show that this transporter also exports ABA from the endosperm (Figs 2 and 5a). Unlike AtABCG31, AtABCG25 seemed to be expressed in the embryo too (Fig. 2b). However, the expression levels of *AtABCG25* in seed tissues were much lower than those of *AtABCG31*, and the embryonic expression level of *AtABCG25* was barely above the background level. Therefore, this result either indicates that *AtABCG25* is not expressed in the embryo or that it is only expressed at low levels. It is possible that low levels of AtABCG25 contribute to the proper distribution of ABA within the embryonic tissues. However, the major site of AtABCG25 function in seeds is most likely the endosperm, where it is expressed at significant levels. The observation that the double knockouts of *AtABCG25* and *AtABCG31* exhibited a stronger phenotype than any of the single knockouts indicates that both transporters are needed to efficiently block germination when GA levels are low and that both transporters exhibit only a limited ABA efflux capacity. In addition, the double knockouts exhibited lower PAC-induced ABA accumulation than the wild type (Supplementary Fig. 6b), suggesting that a defect in ABA secretion may have inhibited ABA biosynthesis by a negative feedback mechanism.

It is interesting to note that AtABCG40, which is broadly expressed in plants, regulates many different physiological properties, including lateral root number, main root growth, seed germination and stomatal movement^40^. These properties can all be explained by the transport of a single substrate, ABA^38^. In contrast, AtABCG30 is specifically expressed in imbibed seeds, and thus its role seems to be restricted to the inhibition of germination. Our data indicate that both AtABCG40 and AtABCG30 contribute to ABA uptake into the embryo and are required to maintain ABA fluxes. This is supported by former data showing ABA transport activity in heterologous systems^40^ and our data demonstrating that AtABCG30-expressing yeast take up far more ABA than the corresponding empty vector control (Fig. 5d). When expressed in yeast, AtABCG40 and AtABCG30 exhibited similar ABA transporting activities (Supplementary Fig. 6f). Since these two genes exhibit different expression patterns *in planta*, the relative contribution of each transporter to overall ABA flux changes depending on the particular tissue and developmental stage. Thus, the transporters involved in ABA release and uptake are not completely redundant, and all four ABC transporters are required to efficiently suppress seed germination. Recently, ABA-importing transporter 1 (AIT1), which is expressed mainly in the vasculature and also in the embryo, was reported to act as an ABA importer for exogenously supplied ABA^42^, raising the question of whether this ABA importer also suppresses embryo growth.

The four *atabcg* mutants studied here are in the Col-0 background, which is among the weakest in dormancy among *Arabidopsis thaliana* ecotypes^16^,^19^,^55^. As a result, we evaluated the roles of the transporters in the absence of GA synthesis, which leads to endogenous ABA accumulation^13^. This is because when seeds are highly dormant, GA is unable to efficiently downregulate DELLA factor accumulation, and the endosperm releases ABA in a DELLA-dependent manner to block embryonic growth^32^. Therefore, it is likely that the AtABCG transporters identified in this study also serve as ABA efflux and influx transporters to block the germination of naturally dormant seeds. However, this conclusion will require further evaluation using *atabcg31*, *atabcg25*, *atabcg30* and *atabcg40* mutant alleles isolated in highly dormant ecotypes.

Most studies on ABA transport have sought to elucidate aspects of the plant's response to environmental stress, such as guard cell closure or long-term adaptation to drought or salinity. Thus, previous discussions on ABA transporters have focused mainly on long-distance transport from the drought-stressed root to the shoot^56^. However, here we have identified four ABA transporters that act in concert to regulate the developmental process of seed germination in adjacent tissues within the seed. These transporters transfer ABA from the site of ABA synthesis (the endosperm) to its target (the embryo) to prevent germination (Fig. 5e). It is plausible that other stress- and development-related responses also require the coordination of short- and long-distance transporters of ABA, in a manner similar to the numerous auxin transporters that collaborate at short and long distances to control plant development.

## Methods

### Plant material and growth conditions

All *Arabidopsis* wild-type plants used in this study were of the *Columbia-0* ecotype. Plants as well as seed coats and embryos were incubated on ½ Murashige–Skoog (MS) agar (0.8%) media (22/18 °C; 16/8 h day/night; 40 μmol m^−2^ s^−1^ light). Experiments were performed with wild-type and mutant plants grown in parallel and seeds were collected at the same time point. Seeds were sown on ½ MS agar (0.8%) media and cultured for 10 days (22/18 °C; 16/8 h day/night; 40 μmol m^−2^ s^−1^ light). Seedlings were transferred to the soil and cultured in a greenhouse (22/18 °C; 16/8 h day/night). Fully developed and ripened brown siliques were collected.

### Seed germination assay

For the germination assay, 12 seeds were placed on a 0.45-μm pore-size nylon membrane on ½ MS medium and incubated at a light intensity of 40 μmol m^−2^ s^−1^ (16/8 h day/night) at 22/18 °C. For the quantification, the seeds with (cold) or without (no cold) pretreatment in the dark at 4 °C for 2 days were sowed on ½ MS medium in the absence or presence of 0.5–1.0 μM ABA. The seeds were incubated under 16-h light and 8-h dark conditions at 22 °C.

### Quantitative real time RT–PCR

Total RNA was extracted from dissected embryos or endosperms, or whole seeds of the Col-0 wild type using the RNeasy Plant Mini Kit (Qiagen). One microgram of total RNA was treated with *DNAse*I (Roche) to remove any residual genomic DNA. cDNA was synthesized with GoScript reverse transcriptase (Promega). qRT–PCR was performed with the SYBR Kit (TAKARA) to detect *AtABCG25*, *AtABCG30*, *AtABCG31*, *AtABCG40*, *AtABI4*, *AtEPA1* and *AtUbiqutin11* transcript levels. Amplified samples were normalized against *AtUbiqutin11* levels. Primer sequences were as follows: for *AtABCG25* (At1g71960), forward (5′-GAGACGCCATGGCTTACTTTGA-3′) and reverse (5′-AATACATGTTGTTATTCCACCGCC-3′); for *AtABCG30* (At4g15230), forward (5′-CATGGCAGTGACTCTACGCTCATCT-3′) and reverse (5′-TTGCCACGATAATGGGATAAGCG-3′); for *AtABCG31* (At2g29940), forward (5′-TCCAAAAGCCTTTGATTCCAGT-3′) and reverse (5′-TCCCACAGGACTTTTTTTATCTTCTC-3′); for *AtABCG40* (At1g15520), forward (5′-CTGAGCGAAAGCATAAGGCATG-3′) and reverse (5′-TGGAGAAATCCTCCTTACACAGCC-3′); for *AtABI4* (At2g40220), forward (5′-ATGGACCCTTTAGCTTCCCA-3′) and reverse (5′-CCACTTGCGAGTGCGCTTAC-3′); for *AtEPR1* (At2g27380), forward (5′-CCACCTGTCAAGCCACCGCCAAT-3′) and reverse (5′-GTAAGTTGGTGTCGGAGGCTTATGCA-3′)^46^; for *AtUbiqutin11* (At4g05050), forward (5′-GAACCAAGTTCATGTATCGT-3′) and reverse (5′-ACACTCATCAAACTAAGC AC-3′) (ref. 57).

### Promoter constructs

The *AtABCG30* promoter::*uidA* reporter gene construct was generated by PCR amplification of a 2.0-kb promoter region of *AtABCG30* from genomic DNA using primers containing *Cla*I and *Xba*I restriction sites (5′-ATCGATTCCACTGGCCAAATTTCTCTATACA-3′ and 5′-TCTAGATTTCAGTTAAAATGCCAACGGAA-3′) and ligation of the PCR product into pBI101.2 (Clontech). The *AtABCG31* promoter::*uidA* reporter gene construct was generated by PCR amplification of a 2.5-kb promoter region of *AtABCG31* from genomic DNA using primers (5′-CTCATGTGGTAGACTGCTAA-3′ and 5′-CTCCATACCAACTCTACGAA-3′) and recombination of the PCR product into the pMDC163 using the Gateway System. All constructs were verified by sequencing.

### Intracellular localization of AtABCG31 and AtABCG30

The *AtABCG30* construct was generated by PCR amplification of a 6.2-kb genomic DNA region of *AtABCG30* from *Arabidopsis* genomic DNA using primers containing *Kpn*I restriction sites (5′-GGGGTACCATGATCCAAACAGGTGAAGAAGATG-3′ and 5′-GGTACCTTTCTTTTGGAAACTGAGTT-3′). The resulting construct was ligated into the pART7 vector^58^. In the case of *AtABCG31*, a previously reported *35Spro::sGFP::AtABCG31* construct^48^ was used. Each construct was introduced into *Arabidopsis* mesophyll protoplasts using the polyethylene glycol method^59^,^60^ along with a plasma membrane marker PM-rK (CD3–1007, ABRE). Protoplasts were isolated from 6-week-old wild-type *Arabidopsis* leaves by enzyme treatments. For each transfection, 200 μl cells (2 × 10^5^ protoplasts per ml) were incubated with 20 μg total DNA and 220 μl 40% (w/v) PEG4000 solution. Seventy hours after incubation under dim-light conditions, green (GFP) and red (mCherry) fluorescence signals were observed and captured using a confocal microscope (Leica TCS SP5).

### Seed coat bedding assay

Detailed protocols on how to assemble SCBA can be found in Lee *et al*.^33^,^34^. For the SCBA, which tests the repressive activity of the endosperm, embryos were dissected from *aba2-1* mutant seeds 4 h after imbibition^33^. Twenty dissected embryos were placed on a layer of 80 seed coat beddings dissected from freshly collected mutant seeds on the surface of 10 μM PAC containing ½ MS agar medium and incubated under 40 μmol m^−2^ s^−1^ light (continuous light) at 22 °C.

To evaluate the embryo's capacity to uptake germination inhibitor from the endosperm, 20 dissected embryos were placed on a layer of 80 seed coat bedding dissected from dormant Cvi seeds placed on ½ MS agar medium and incubated at a light intensity of 40 μmol m^−2^ s^−1^ (continuous light) at 22 °C.

For the embryonic growth assay of dissected embryos, 12 embryos dissected from wild-type (Col-0), *atabcg* mutant seeds or whole seeds were placed on a 0.45-μm pore-size nylon membrane on ½ MS medium in the absence or presence of 0.1 μM ABA. In this experiment, we used a lower ABA concentration, since exogenously added ABA inhibits the growth of dissected, coatless embryos much more effectively than it inhibits the growth of embryos in whole seeds.

### Assay of secreted and remaining ABA levels

Endosperms and testa were dissected from 500 seeds of the wild type and *atabcg25* and *atabcg31* single-knockout and *atabcg25 atabcg31* double-knockout mutants imbibed for 24 h on ½ MS agar media complemented with 10 μM PAC or for 1 h on the same medium without PAC. To collect excreted ABA, the tissues were subsequently incubated in 100 μl ½ MS liquid medium without PAC for 24 h. After incubation, the remaining tissues were washed with ice-cold ½ MS medium. ABA was extracted with 80% methanol solution, vacuum dried and the pellet was resuspended with Tris-buffered saline buffer. ABA levels were determined using the ELISA Kit (Agdia)^61^.

### ^3^H-ABA accumulation assay

Fifty dissected embryos were incubated in 100 μl of a bathing solution (pH 6.5) containing 12.5 nM ^3^H-ABA (1.63 Tbq mmol^−1^) and 17.5 pM ^14^C-glycerol (5.40 GBq mmol^−1^) for 30 min at room temperature. After incubation, embryos were washed three times with 250 μl ice-cold bathing solution, and their radioactivity was determined by liquid scintillation counting.

### Heterologous expression of AtABCGs and ABA loading assays

*AtABCG31* cDNA was cloned into the *Not*I and *Xba*I sites of pYES2NT/C (Invitrogen). *AtABCG30* cDNA was cloned into the *Kpn*I and *Xba*I sites of pYES2NT/C. All constructs were verified by sequencing. AtABCGs expressing yeast lines were generated using the electroporation method and selected on uracil-minus minimal media. ^3^H-ABA-loading tests were performed as described previously with minor modifications^40^. Cells were cultured in minimum salt–galactose medium in the absence of uracil (SG-URA), supplemented with 1.0 % raffinose, at pH 6.5, and collected by centrifugation at the mid-log phase (up to OD_600_=1.0). Yeast cells were washed two times using SG-URA medium and subsequently resuspended in reaction buffer (1 mM KCl, 1 mM MES (2-(N-morpholino) ethanesulfonic acid), 20 mM glucose, at pH 6.5) at an OD_600_=10. ^3^H-ABA (50 nM, 7.4 kBq, 1.63 TBq mmol^−1^) was then added to the cell suspension and gently mixed. At the times indicated, 100 μl of cell suspension was filtered through nitrocellulose membranes, and the cells remaining on the filter were washed two times with 2 ml of ice-cold water. The radioactivity on the filter was determined using a liquid scintillation counter.

## Additional information

**How to cite this article:** Kang, J. *et al*. Abscisic acid transporters cooperate to control seed germination. *Nat. Commun*. 6:8113 doi: 10.1038/ncomms9113 (2015).

## Supplementary Material

Supplementary Information Supplementary Figures 1-7 and Supplementary References
