# Supplementary material for: Abscisic acid transporters cooperate to control seed germination
Source: Nat Commun. 2015 Sep 3;6:8113. doi: 10.1038/ncomms9113 (PMC4569717; doi:10.1038/ncomms9113)
Supplement: Supplementary Information — Supplementary Figures 1-7 and Supplementary References [file ncomms9113-s1.pdf]

Supplementary Information

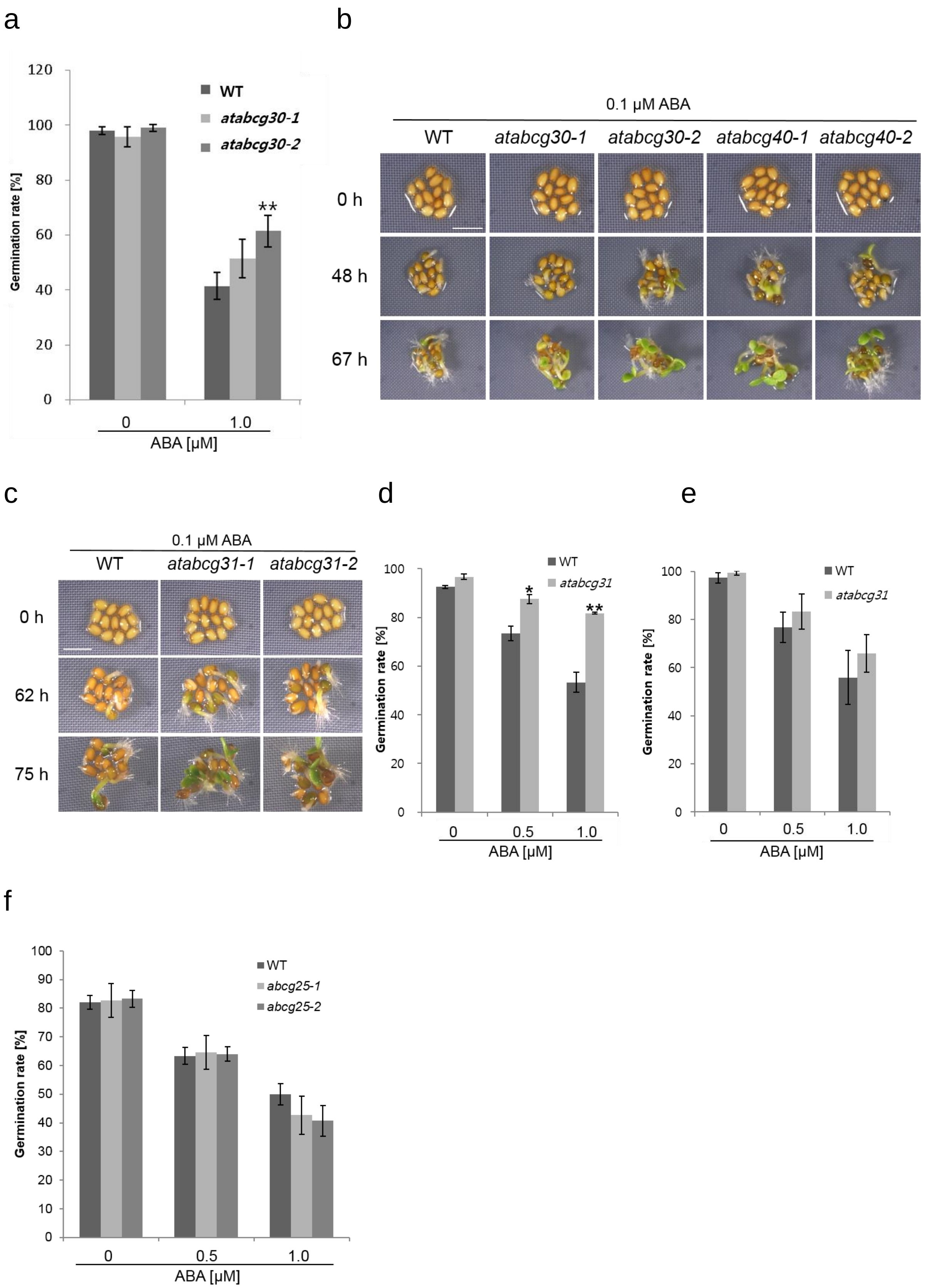

Supplementary Figure 1. *atabcg30, atabcg31,* and *atabcg40* mutant seeds germinate faster than the wild type on ½ MS medium supplemented with ABA (**a** and **d**-**f**) Germination of wild-type and *atabcg* mutant seeds on ½ MS medium supplemented with or without 0.5 or 1.0 μM ABA. The control medium was not supplemented with ABA (0 ABA) and contained the same amount of DMSO used to make 1.0 μM ABA solution. The seeds were placed in the dark at 4°C for 2 days, sowed, and incubated under 16-h light and 8-h dark conditions at 22°C. The percentage of germinated seeds was determined 36 h after sowing by scoring the number of seeds with radicle extrusion (**d**) or 5 days after sowing by scoring the number of fully expanded green cotyledons (**a**, **e**, and **f**). Data are means ± s.e.m. from three independent experiments (*N = 3, n = 4 x 100 seeds*). (*$p<0.05$, **$p<0.01$ compared with the wild type by Student's *t*-test). (**b**, **c**) Seed germination on ½ MS medium containing 0.1 μM ABA. Seeds were incubated under continuous light conditions at 22°C. Results shown are representative of three independent experiments. Similar results were obtained in all experiments. Scale bar = 100 μm.

a

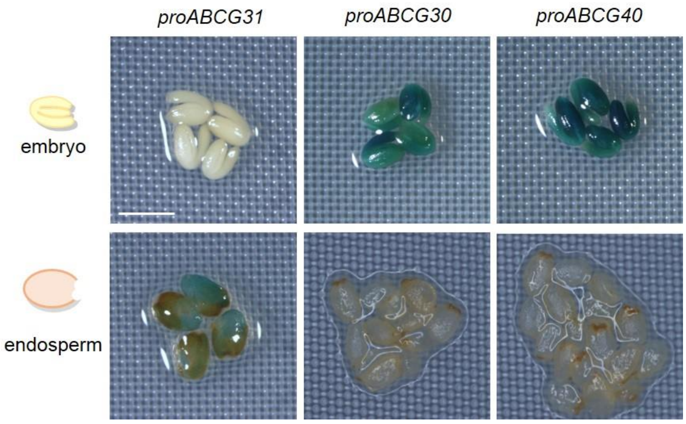

b

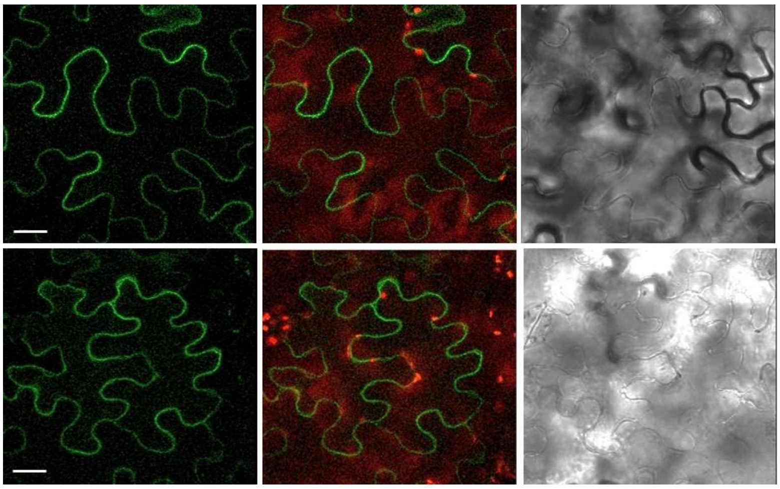

Supplementary Figure 2. *AtABCG31* is expressed mainly in the endosperm, whereas *AtABCG30* is expressed specifically in the embryo (**a**) *AtABCG31* promoter-GUS (*pAtABCG31::uidA*) reporter gene activity was detected only in the endosperm, and not in the embryo (left). *AtABCG30* promoter-GUS (*pAtABCG30::uidA*) and *AtABCG40* promoter-GUS (*pAtABCG40::uidA*) activities were detectable only in the embryo, and not in the endosperm (middle and right). These data replicates of those presented in Figure 2g, but obtained with independent transgenic lines. Embryos (upper panel) and endosperms (lower panel) were dissected from mature seeds and incubated in GUS solution for 30 min. Representative results out of 8 (*AtABCG30*), 12 (*AtABCG31*), and 9 (*AtABCG40*) different T3 promoter-GUS lines are shown. Scale bar = 100 μm. (**b**) Plasma membrane localization of AtABCG31 (upper panels) and AtABCG30 (lower panels) in tobacco epidermis cells. The sGFP::*AtABCG31* construct[1] was previously reported by

Choi *et al*. (2014). The *AtABCG30* construct was generated by PCR amplification of a 6.2 kb genomic DNA region of *AtABCG30* from *Arabidopsis* genomic DNA using primers containing *Xba*I and *Sca*I restriction sites (5’-TCTAGAATGATCCAAACAGG TGAAGAAGATG-3’ and 5’-AGTACTCAATTTCTTTTGGAAACTGAGTTTGCT-3’) and 700-bp sGFP from 326 sGFP vector using primers containing *Sca*I and *Sac*I restriction sites (5’-AGTACTATGGTGAGCAAGGGCGAG-3’ and 5’-GAGCTCTTACTTGTACAG CTCGTCCATGC-3’). The resulting construct was ligated into the pBI121. The two constructs were introduced into *Agrobacterium* strain GV3101 and inoculated to *N. benthamiana* leaves with *Agrobacterium* strain RK19[1]. Forty eight h after inoculation GFP fluorescence signals were observed and captured using a confocal microscope (Leica TCS SP5). The left panels show GFP fluorescence, the middle panels show the merged images of GFP and chlorophyll auto-fluorescence, and the right panels show bright-field. Scale bar = 20 μm

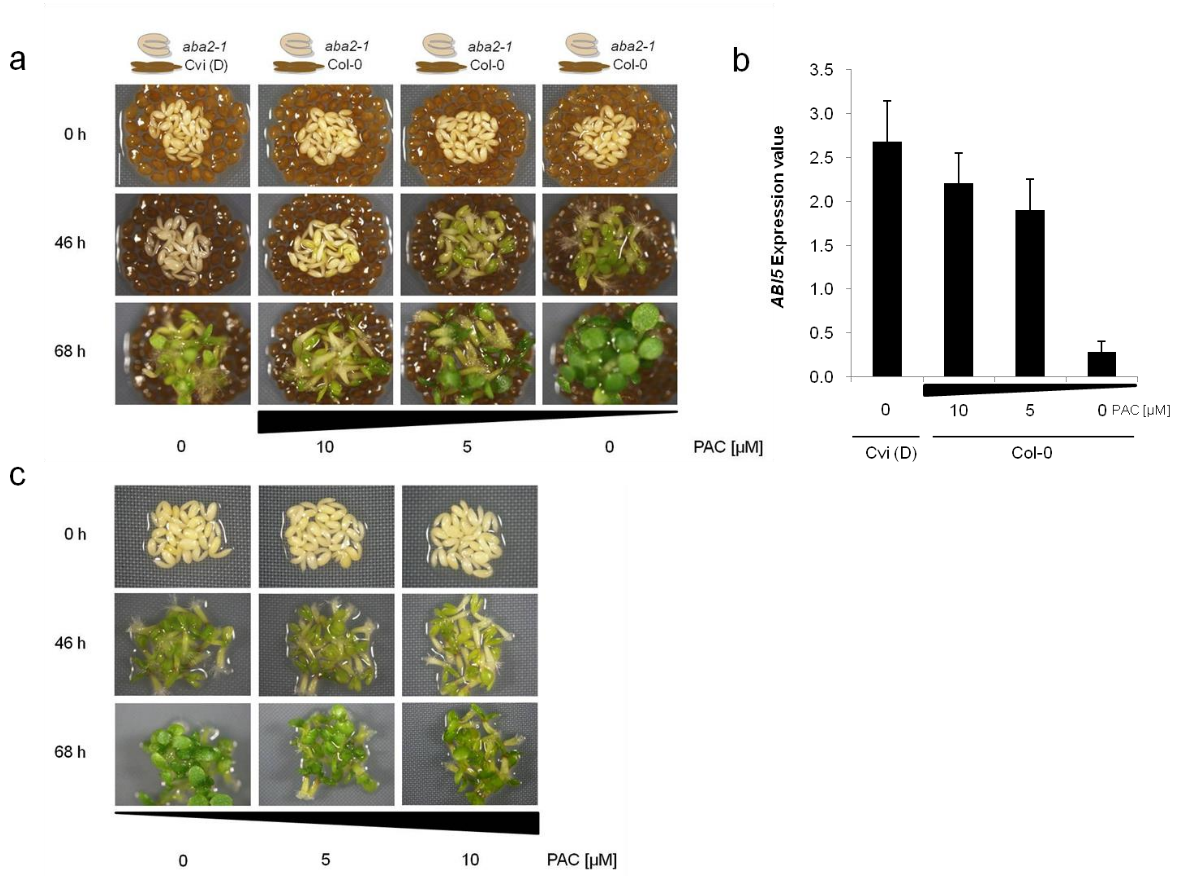

Supplementary Figure 3. Treatment with PAC, an inhibitor of GA biosynthesis, induces non-dormant Col-0 seed coats to behave similarly to the strongly dormant Cvi seed coats in a SCBA while not altering the development of dissected embryos (**a**) Germination repression activity of the seed coat beddings of Cvi (D) and Col-0 on media containing 0, 5, and 10 μM PAC. Embryos dissected from *aba2-1* mutant seeds were placed on a layer of seed coats dissected from Cvi or Col-0 seeds. Photographs were taken at 0 h to 68 h after imbibition. Note that the non-dormant Col-0 seed coat beddings treated with 10 μM PAC were similar to the strongly dormant Cvi (D) seed coat beddings in terms of their ability to repress growth of *aba2-1* embryos. Scale bar = 100 μm. (**b**) q-PCR analysis of *ABI5* transcripts. q-PCR was performed using total RNA isolated from Cvi (D) and Col-0 seeds incubated on ½ MS agar media containing 0, 5, and 10 μM PAC. Data were normalized using *Ubiquitin11*. Data are means ± s.e.m. from three independent experiments ($N = 3, n = 2$). (**c**) The PAC treatment did not affect the development of the dissected embryos. Embryos dissected from *aba2-1* mutant seeds were placed on a serial concentration of PAC-

containing media. (**a**, **c**) Results shown are representative from three independent experiments. Similar results were obtained in all experiments. Scale bar = 100 μm.

a

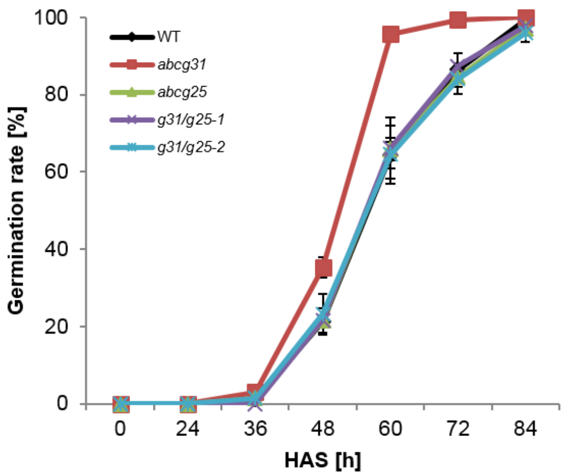

b

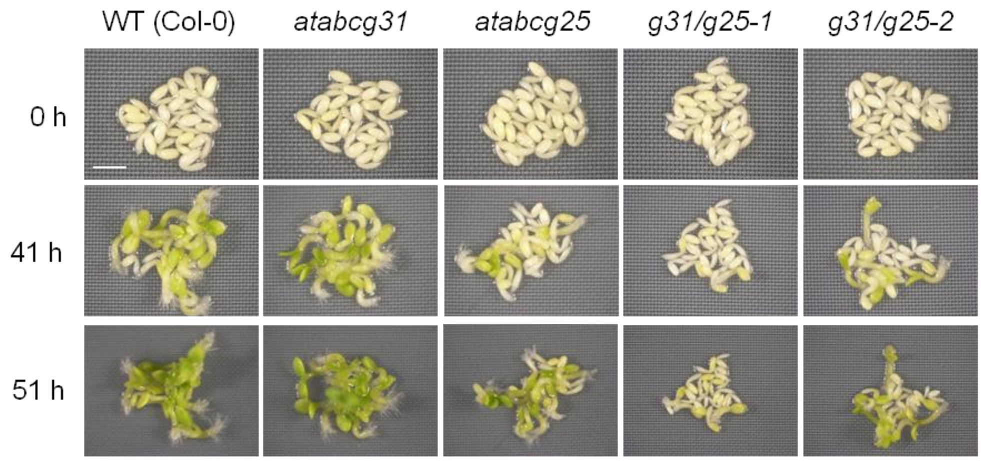

Supplementary Figure 4. Whole seeds and isolated embryos of *atabcg25* mutants differ in germination related phenotypes Isolated embryos of *atabcg25* single knockout or *atabcg31 atabcg25* double knockout seeds germinate more slowly than do those of the wild type (**b**), whereas the whole seeds of the mutants do not exhibit such a difference (**a**). (**a**) Germination of whole seeds on ½ MS medium without stratification. The percentage of germinated seeds was determined by scoring the number of seeds with radicle extrusion. Data are means ± s.e.m. from three independent experiments *(N = 3, n = 4 x 100 seeds*). (**b**) Growth and the subsequent development of isolated embryos placed on ½ MS medium. The seeds tested are from the wild type (Col-0), *atabcg31*, *atabcg25*, and *atabcg31 atabcg25.* A representative out of three independent experiments giving rise to similar results is shown. Scale bar = 100 μm.

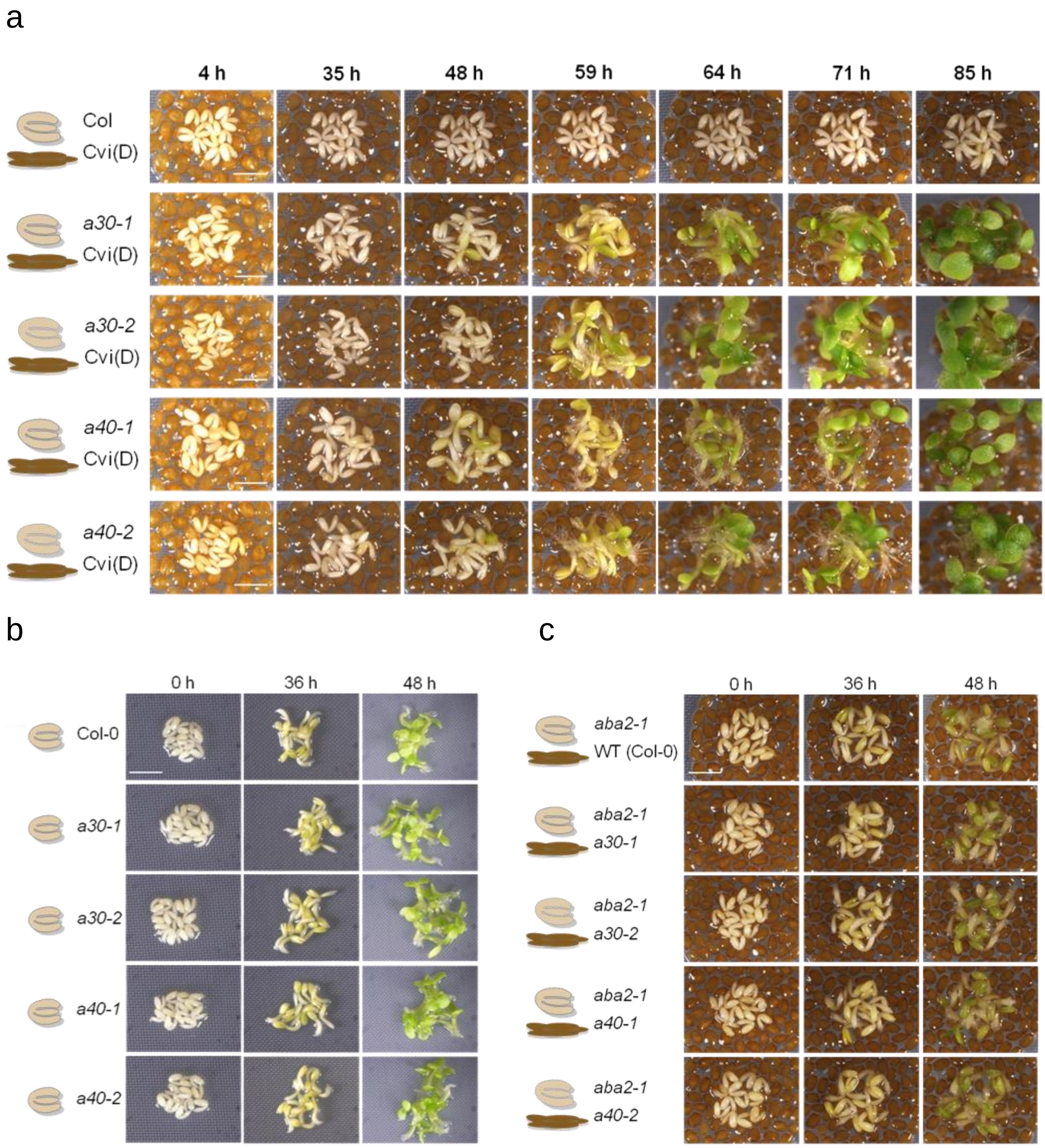

Supplementary Figure 5. Seed coat bedding assays to characterize the functions of AtABCG30 and AtABCG40 (**a**) *atabcg30* and *atabcg40* mutant embryos germinate faster than the wild type on seed coat bedding derived from Cvi (Cvi (D)). Embryos dissected from wild-type (Col-0) and *atabcg* mutant seeds were placed on a layer of seed coat beddings dissected from dormant Cvi seeds (Cvi (D)). Photographs were taken 0 h to 85 h after imbibition. The dissected embryos of *atabcg30* and *atabcg40* are less sensitive than those of the wild type to the inhibitory molecules

secreted from the seed coat beddings of Cvi (D). (**b**) Embryos dissected from *atabcg30* and *atabcg40* exhibit growth and greening rates similar to those of the wild type (Col-0) and *atabcg* on ½ MS medium. (**c**) Endosperm derived from *atabcg30* and *atabcg40* did not affect the development of *aba2-1* embryos. Embryos dissected from *aba2-1* mutant seeds were placed on a layer of seed coat beddings dissected from WT (Col-0), *atabcg30* (*a30-1* and *a30-2*), and *atabcg40* (*a40-1* and *a40-2*) seeds. Photographs were taken 0 h to 48 h after imbibition. Results shown are representative from three independent experiments. Similar results were obtained in all experiments. Scale bar = 100 μm.

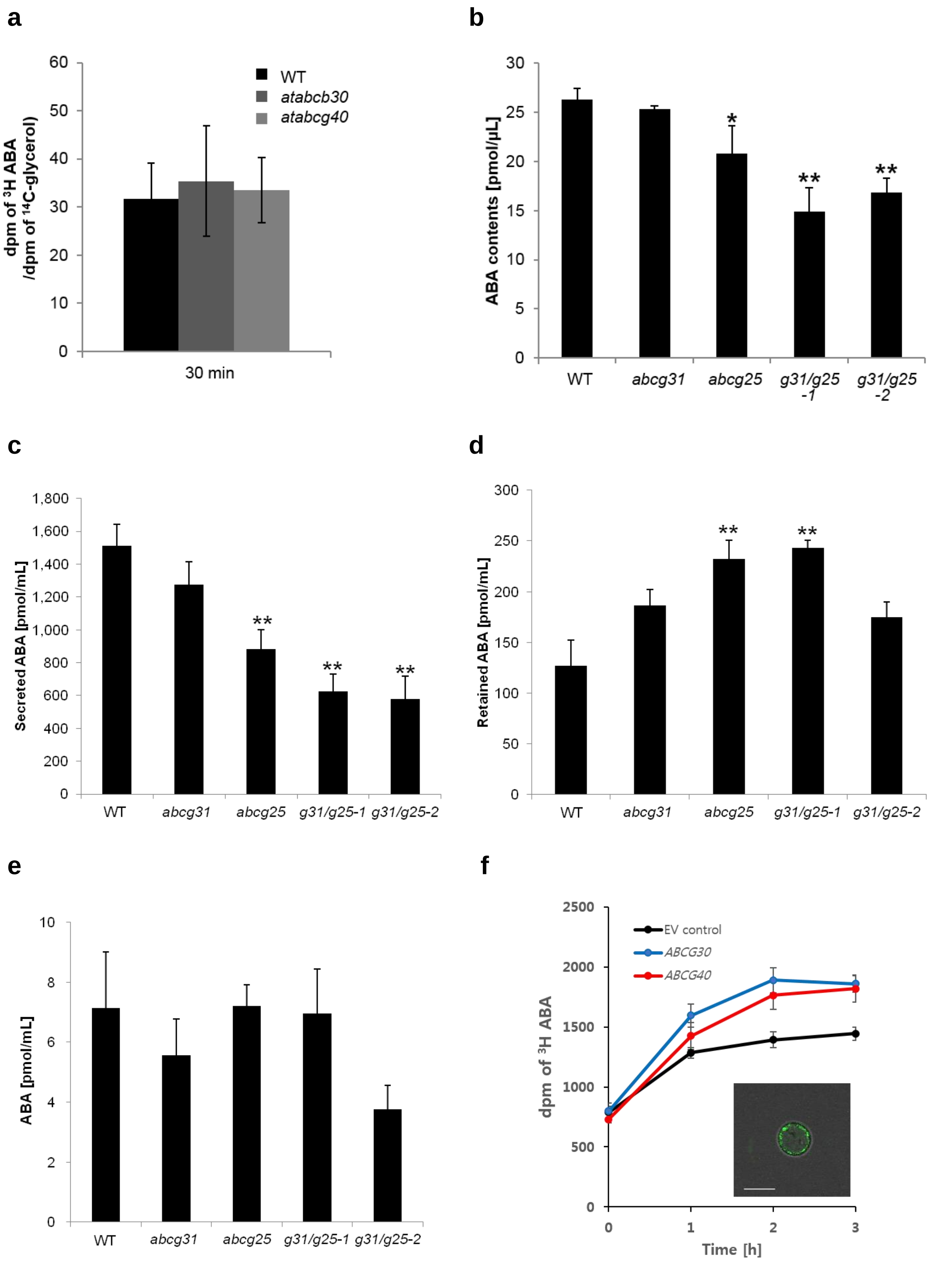

Supplementary Figure 6. ABA contents in *Arabidopsis* seeds and transport activity of AtABCGs (**a**) Accumulation of ^{3}H-ABA by embryos dissected from the seeds of various genotypes. Embryos were incubated in betaine buffer containing 12.5

nM ^{3}H-ABA (1.63 Tbq/mmol) and 40 pM ^{14}C-glycerol (5.40 GBq/mmol) at pH 6.5 on ice for 30 min. Radioactivity counts of the embryos were normalized to the ^{14}C-glycerol dpm (disintegrations per minute) value. Data are means ± s.e.m. of $n = 4$ from two independent experiments. ^{3}H-ABA uptake was similar for the dissected embryos of the wild type, *atabcg30*, and *atabcg40*. (**b**) Endogenous ABA contents in whole seeds. Wild-type, *atabcg31*, *atabcg25*, and *atabcg31 atabcg25* (*g31/g25-1, g31/g25-2*) seeds were incubated on medium containing 10 μM PAC for 24 h before the ABA content was determined using an ELISA Kit (Agdia). (**c**-**d**) The tissues surrounding the embryo of mutants release less ABA than do those of the wild type. This experiment was carried out in the same way as described for figure 5a and b, except that these seeds were not treated with PAC. The tissues surrounding the embryos of five hundred wild-type, *atabcg31*, *atabcg25*, and *atabcg31 atabcg25* (*g31/g25-1, g31/g25-2*) seeds were dissected 24 h after imbibition on ½ MS medium. The tissues were then incubated in ½ MS liquid medium for 24 h before the ABA content was assayed using an ELISA Kit (Agdia). The aqueous medium was collected for assay of secreted ABA (**c**), and the remaining tissues were washed three times and extracted to assay the retained ABA (**d**). (**e**) Endogenous ABA contents in whole seeds (not treated with PAC). Wild-type, *atabcg31*, *atabcg25*, and *atabcg31 atabcg25* (*g31/g25-1, g31/g25-2*) seeds were incubated on ½ MS medium for 1 h before the ABA content was determined using an ELISA Kit (Agdia). Data are means ± s.e.m. of $n = 6$ (**b**-**d**) or $n = 9$ (**e**) from three independent experiments (*$p<0.05$, **$p<0.01$ compared with the wild type by Student's *t*-test). (**f**) Time-dependent loading assay of ^{3}H-ABA using yeast cells expressing AtABCG40, AtABCG30 or transformed with the empty vector (EV). Yeast cells were incubated in SG-URA medium containing 50 nM ^{3}H-ABA (7.4 kBq, 1.63 Tba/mmol) at pH 6.5. Data are means ± s.e.m. of $n = 12$ from three independent experiments ($N = 3, n = 4$). ***Inset***: Plasma membrane localization of AtABCG30 in yeast. To generate the *AtABCG30*::GFP-6 construct, a 700-bp *GFP-6* fragment was PCR amplified from pART7 using primers containing *Xba*I restriction sites (5'-TCTAGAATG AGTAAAGG AGAAGAACTT-3' and 5'-TCTAGACTACTTGATCAGCTCGTCCATGCC-3'). The *GFP-6* fragment was then ligated into the pYES2NT/C::AtABCG30 construct. The construct was introduced into yeast cells by electroporation. Cells were cultured in minimum salt-galactose medium in the absence of uracil (SG-URA), supplemented with 1.0 % raffinose, until reaching a density of $OD_{600} = 0.2$. GFP fluorescence signals

were observed and captured using a confocal microscope (Leica TCS SP5). The inset is a merged GFP fluorescence and bright-field image. Scale bar = 5 μm.

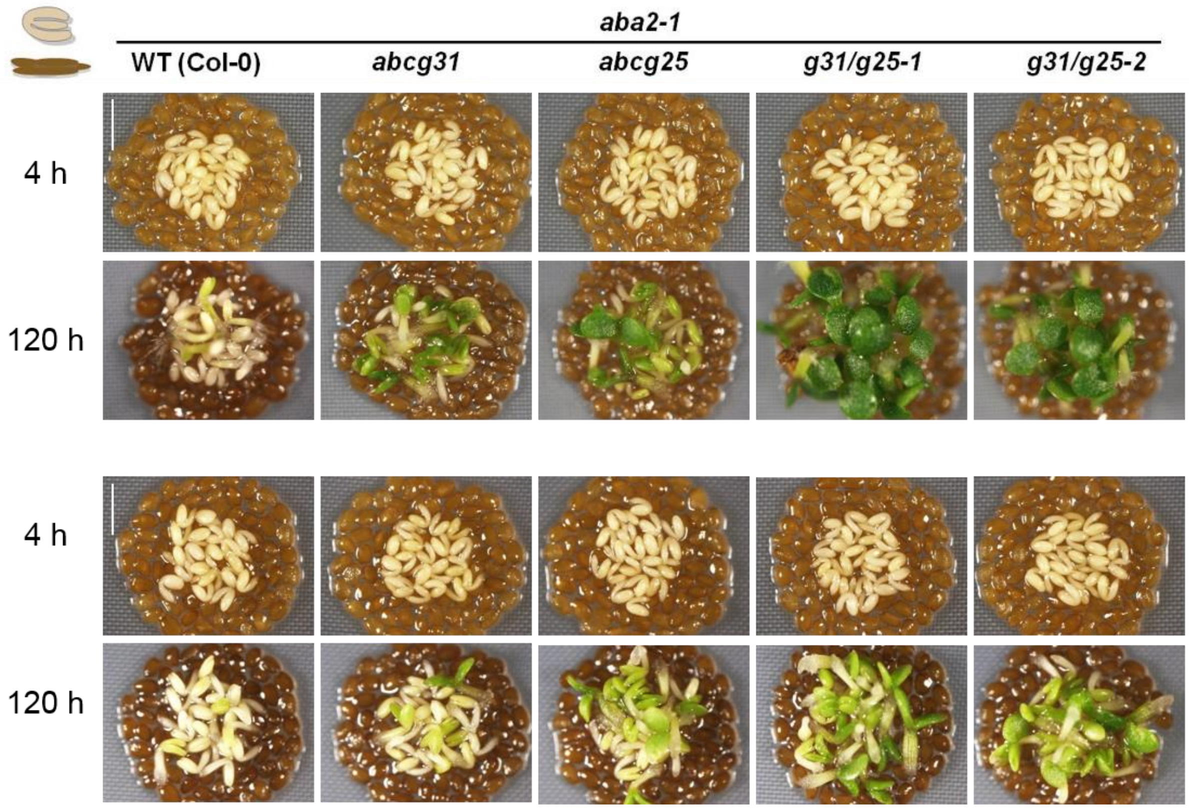

Supplementary Figure 7. Seed coat beddings of *atabcg31* and *atabcg25* single mutants and *atabcg25 atabcg31* double mutants repressed the embryonic growth of *aba2-1* less effectively than did those of the wild type Embryos dissected from *aba2-1* mutant seeds were placed on a layer of seed coat beddings dissected from the wild type (Col-0), *atabcg31*, *atabcg25*, and *atabcg31 atabcg25* (*g31/g25-1*, *g31/g25-2*). These data are replicates of those presented in Figure 3e, but obtained with independent experiments. Scale bar = 100 μm.

Supplementary Reference

1. Choi, H. *et al*. The role of *Arabidopsis* ABCG9 and ABCG31 ATP binding cassette transporters in pollen fitness and the deposition of steryl glycosides on the pollen coat. *Plant Cell* **26**, 310-324 (2014).
